# Hospital and Institutional News

**Published:** 1918-01-26

**Authors:** 


### HOSPITAL AND INSTITUTIONAL NEWS.

### HOSPITAL TREATMENT OF DISCHARGED SOLDIERS.

ON November 24, 1917, page 155, we announced the appointment of the Rev. G. B. Cronshaw and Mr. E. W. Morris, as representatives of the British Hospitals Association, to confer with the War Office authorities in reference to payments for the hospital treatment of sick and wounded sailors and soldiers. A report on this question has been submitted to Sir Charles Harris, Financial Secretary to the War Office, and the representatives of the Association are now awaiting the summons to a conference. The deferred deputation to the Ministry of Pensions was received on December 20 last. This resulted in the formation of a Joint Committee, representing the Ministry and the British Hospitals Association, to discuss the subject of payment for the hospital treatment of discharged soldiers. As a result we have the satisfaction to announce that a basis of payment has been agreed to, the details of which will, it is expected, be ready to be issued on or about January 25, with an explanatory statement and recommendation to the hospitals from the Council of the British Hospitals Association. Our readers will be gratified to learn that the suggested payment is on the basis of a differential rate for different classes of hospitals, and that the rate will be found to be an advance on the 4s. per diem at present allowed by the War Office.

### CAPTAIN TUNNARD'S SUCCESSOR.

THE vacancy in the office of secretary to King's College Hospital, with which we dealt in our issue of January 5, page 281, has been filled by the appointment of Mr. Richard J. Coles, who for the last thirteen years has held the post of secretary-superintendent to Addenbrooke's Hospital, Cambridge. Mr. Coles' experience in the hospital world has been considerable. His first secretaryship was at the Warneford Hospital, Leamington, whence he proceeded to Bristol Royal Infirmary as secretary and house governor. On Mr. Coles' appointment to Cambridge thirteen years ago we ventured to express our expectation that he would continue his successful career at Cambridge to the satisfaction of his committee and the great advantage of the hospital, whose interests he would serve. This forecast has been happily fulfilled. He has reorganised many things, including the secretary's office and accounts, and several other matters material to the successful management of a hospital. Important reconstruction and extensions have been carried out. They include an electrical and *x*-ray department, clinical laboratories and the pathological department, new wards for children, and an interesting and attractive new out-patient department and dispensary. In addition to these a new boiler-house, pump-rooms, and hot water installations were completed in 1915. The financial position of the hospital, on the figures, has been improved, the total ordinary income in 1904, the year before Mr. Coles joined, having been £7,851, whilst in 1915 it amounted to £11,394. These figures may be regarded as indicative, though not normal, owing to the war. In this connection our readers will remember the article entitled "Income by the Hundredweight," which we published in our issue of the 12th instant, page 314. Mr. Coles is there shown to have developed Pound Day for the county hospital into an organisation yielding substantial gifts from every household in a village, the gifts in one year from four villages having weighed about forty tons and being equal, with the cash gifts, to £350 at least. The post vacated by Mr. Coles at Addenbrooke's Hospital is advertised in our columns this week. The salary offered is £350 a year, with a house or an allowance for a house.

### RESTRICTED HOTEL MEALS.

THE limitation of public meals which has just been announced is striking proof that the authorities recognise that the time has come to curtail the amount of food consumed in hotels and restaurants, in order to conserve our food supplies and so render them sufficient for our needs. The observance of two meatless days in the week should make a perceptible difference in the amount of meat consumed, and the same effect will be produced by the order that no meat is to be served with breakfast. Afternoon tea is also to be restricted, for the amount of solid allowed is only an ounce and a half, and guests have to bring their own sugar. Public eating-houses which agree not to serve any meal exceeding 1s. 2d. in price are not to be bound by certain of these restrictions, and need not observe the meatless days. Boarding-houses with more than five bedrooms for boarders have to obey all these rules, but they are not to apply to private houses, at all events at present. It is perhaps the difficulty of seeing that the regulations are obeyed that prevents their application to private houses, though the restrictions in the power of purchasing supplies may be considered a sufficient deterrent.

### HONOUR TO A MEDICAL HERO.

THE history of the medical profession contains many records of signal devotion to duty, but few, we think, will be found to equal that of Major Priestly, who has been awarded the Gold Medal of the West London Medico-Chirurgical Association. When the typhus epidemic broke out in the spring of 1915 in the Wittenberg internment camp the principal medical officer, Dr. Aschenbach, fled, and his colleagues followed suit. Major Priestley filled the gap, and although the prisoners' condition was so deplorable that there was not even a clinical thermometer in the whole camp, he succeeded in organising nursing arrangements, and was instrumental in saving many lives. He and his orderlies toiled on unremittingly till he himself fell sick and many of the volunteer nurses themselves did. The Germans behaved throughout with almost incredible brutality. Prisoners were fired at and wounded by their guards if they showed their faces at the windows or doors. The only place of safety was upon the floor of the huts, and even here five men were hit by rifle volleys. The food was appalling, the men scantily clad, sanitation non-existent. Under these conditions the epidemic spread like wildfire. At last Dr. Priestley's example shamed the authorities into action, and some tardy measures were taken to stem the disease. If further proof were needed by any of the true tendencies of "Kultur," the story of the horrors of Wittenberg camp supplies it in abundance.

### HOW TO REDUCE THE NUMBER OF VACANT MILITARY BEDS.

It is a relief to know, after the hesitation of the past year, that the Norfolk and Norwich Hospital has decided to continue the payment of forty guineas a month to the Jenny Lind Infirmary, which by taking over children's cases allowed the Norfolk Hospital to receive wounded, and incidentally the Government grant. The military beds have been more regularly occupied during the past quarter, in consequence of which the financial prospect is less doubtful than it was, though a deficit has still to be expected. Sir Eustace Gurney, at the recent quarterly meeting, aptly said that the instinct of the governors in continuing the payment to the Jenny Lind Infirmary had been fully justified. When the case was under consideration we summarised the position, and it was plain that the fair and proper thing to do was to continue the grant. It is, in fact, a debt for a definite service. Till lately, however, the military authorities did not make it easy for the Norwich Hospital to support its obligation of honour, for till September last the average daily number of unoccupied military beds had been about 100. It has been reduced to thirty during the past quarter by two simple reforms. The first is a system for making a return of vacant military beds to the War Office, and, in conjunction with this, the arrival of smaller convoys at shorter intervals. This plan might be generally followed with advantage.

### THE NEED FOR A NEW POLICY.

It is only since September last that the Royal Hospital, Sheffield, has been receiving a Government grant in respect of the military patients. The financial position is serious, since the interest on the overdraft alone amounts to over £2,000 a year. An appeal for £50,000 is inevitable, but we believe that the way might be prepared by asking for the sum of £8,500, at which the services of the hospital to the wounded before the grant was received are estimated. It is noteworthy that the deficit at the end of the year is expected to amount to almost the same sum. To appeal for a deficit is not very inspiring, but it should be less difficult to ask for the sum incurred on behalf of the wounded. We are not aware that the organisation of sectional subscriptions among every class served by the hospital has been extended lately in Sheffield. We do not know that employers there have been asked to substitute a casual cheque for a definite sum based upon the number of their employees. Yet these methods have been productive in connection with the North Staffordshire Infirmary, and also at Cardiff. So serious a financial burden requires, in our opinion, something more than an appeal to raise it. A series of new organisations of subscriptions is the most promising policy.

### FEWER BOARD MEETINGS.

At a special court of governors of the Royal Berkshire Hospital it has been decided to reduce the number of board meetings to two a month. Previously the board has met once a week, in consequence of which several of those who have been asked to join the board have declined on the ground that they could not afford the time to attend. This action is preferable to the carelessness of those who accept nomination to committees and then rarely put in an appearance, and, like all straightforward actions, has led to a change of policy which seems to be unanimously approved. The rules still permit urgent questions to be brought before the board, but the change would not have been unanimously agreed to if it had not also been advocated as a war measure. The idea is that now the board meetings will be better attended, and that it will be easier to admit the right kind of person on the board.

### THE VALUE OF HOSPITAL VISITORS.

A powerful tribute to the value of intelligent visitors was lately paid by Mr. F. Hatch at a meeting of the Hospital Saturday Governors of North Staffordshire Infirmary. He mentioned his own past efforts to have some of the Saturday Governors placed upon the visitors' list. He asked those present to instruct their representatives to see that several names were added to the visitors' list at the annual meeting. All could not be members of the General Committee; those on the visitors' list could become fully acquainted with the management of the infirmary. This visiting is, indeed, as much an education to the visitors and their friends as a safeguard against mismanagement. It fosters and extends interest in the institution, and is probably most useful in those districts where a large factory population supports on a big scale the Hospital Saturday Fund. Moreover, working class supporters are apt to change their representatives on committees, which often means that almost as soon as a man has learnt committee work he is replaced by a novice; some means of educating the rank and file without unduly swelling the size of committees has to be found. Visitors' lists help to bring this about, and are therefore administratively useful.

### THE LEEDS MEDICAL SCHOOL.

The retirement of Professor de Burgh Birch from the Chair of Physiology at Leeds University is leading to an important change. The teaching of physiology has been divided, and a new chair has been created. Dr. S. Raper has been appointed Professor of Physiology and Bio-Chemistry, and Dr. C. Lovatt Evans is the new Professor of Experimental Physiology. The department of organic chemistry at Leeds University has been investigating the antiseptic powers of various substances containing active chlorine, in consequence of which the two antiseptics chloramine-T and dichloramine-T were introduced. Under the new arrangement further research laboratories will be required, together with a re-equipment of the physiological department. In the bacteriological department of the Leeds Medical School changes are also impending, but their nature has not yet been made public.

### A GRACEFUL GIFT.

Hospital debts, being almost as plentiful as blackberries, do not as a rule weigh heavily upon the minds of honorary, or even official, administrators. There are, however, exceptions to this dictum, and here and there are found those who accept responsibility in a serious light. Mrs. Yerburgh, of Woodfold Park, Blackburn, is such an one, and, as widow of the late president of the Blackburn and East Lancashire Royal Hospital, expresses, as she states in a letter to the secretary, that "it would be a pleasure to me to feel that at the termination of my husband's presidency all was in order." This generous lady, in order to achieve this condition, has sent the hospital a cheque for £3,378, the amount of the deficit at the date of her husband's death. Having in this very practicable and acceptable way wiped off old scores, Mrs. Yerburgh shows her continued personal interest in the institution so well served by Mr. R. A. Yerburgh by becoming an annual subscriber of £250, which sum will be continued during her lifetime so long as the infirmary is maintained by voluntary subscriptions.

### THE FINANCES OF EDINBURGH ROYAL INFIRMARY.

An amusing example of the way in which figures can be represented satisfactorily even in unfortunate circumstances has been provided at Edinburgh. The contributors to the Royal Infirmary are asked to congratulate themselves because, though income and expenditure have alike increased enormously, the deficit is only £1,000 more than it was before the war. Unfortunately, this does not alter the fact that the annual deficit is a very large sum. English observers have long contemplated with wonder the great indebtedness of the Edinburgh and Glasgow hospitals, which no financial policy seems able to abolish. Legacies from time to time tide over the worst evils, but there is nothing like an adequate income. The present proposal is to increase the representation of the working classes on the board of the Royal Infirmary, in the hope that by so doing the working classes in the city may subscribe as generously as those without. The time will come, however, when much greater effort will have to be made, for the discrepancy has long been very serious. In the past year alone the excess of expenditure reached the enormous total of over £26,000.

### THE POWER OF THE PEN.

In consequence of Mr. Rea's appeals on behalf of King Edward VII.'s Hospital, Cardiff, the variety and ingenuity of whose letters have been regularly noted in these columns, the necessary £8,000 has been subscribed, and a deficit on the year's working has been averted. A special vote of thanks was given to Mr. Rea at a recent board meeting. His pen will probably again be enlisted in the big task ahead, whereby it is hoped to increase the number of beds by 180, to abolish, or at least curtail, the list of 1,000 patients now waiting admission. The project for a new nurses' home has a direct bearing on the latter point, for, once it has been provided, a large part of the present buildings will become available for wards. So much has been achieved by General Lee, Colonel Bruce-Vaughan, and Mr. Rea that this task, too, will be accomplished.

### THE DISABLED SOLDIER IN BRISTOL.

It is hoped that an orthopædic training centre may be established at the Cossham Memorial Hospital, Bristol. At a special meeting of the Bristol War Pensions Committee, which has undertaken to urge on the War Office the establishment of a local scheme for the orthopædic treatment of disabled men, Dr. E. H. Cook said that in Bristol about 1,000 patients needed this treatment. Such a patient should be under skilled supervision from the day he entered hospital till his discharge to civil life. Because of the pressure on the military hospitals, Dr. Cook remarked that men were discharged somewhat sooner than would formerly have been the case. A long stay in a properly equipped hospital was therefore necessary. Major Hey Groves said that at present many men were discharged from hospital before they were fit for civil life, and once discharged under existing regulations they could not be readmitted to a military hospital. At Cossham 100 cases could be accommodated, and its use, he thought, could only be temporary, pending a much larger scheme. Between the Ministry of Pensions, which is responsible for discharged men and has no discipline over them in cases where they are slack in attending training centres, and the War Office, which is loath to admit a faulty diagnosis and a premature discharge, these cases are not properly provided for. Hence the urgent need in Bristol for a voluntary scheme, such as the War Pensions Committee has been led to put forward.

### BIRMINGHAM CHILDREN'S HOSPITAL.

Mr. Harold F. Shrimpton, at present assistant secretary at the General Hospital, Birmingham, has been appointed secretary to the Children's Hospital, Birmingham, in succession to Mr. Albert Hastings, who has received an appointment under the Food Production Department, London.

### TRIBUNALS AND TUBERCULOSIS.

The low medical categories of the bulk of the men now being dealt with by the Tribunals show how well, in London at least, the needs of the Army have been made paramount, whilst the trading and manufacturing community has been reduced to the lowest possible ebb, and has to be content with men who are practically useless for the Army, and are of more value in civil life than they would be in some of the subsidiary services in the Army. Very often a Tribunal may deal with fifty cases in a morning, and not find a solitary man fit for general service amongst them. Lately there has been an ever-increasing number of men suffering from tuberculosis before Tribunals. As a rule when a man brings a certificate, or perhaps two, from medical practitioners certifying that he is a patient afflicted with tuberculosis, the Tribunal will refer him to their own tuberculosis officer for confirmation of this, and this has been hitherto sufficient evidence to grant the man a long period of exemption, as it is generally agreed that the Army does not require men suffering from tuberculosis within its ranks. More conclusive evidence is now required ere a man can get this exemption, for the matter has been taken from the Tribunals and left entirely to the National Service Medical Boards to deal with. As a general rule this entails a much longer period of waiting than under the old procedure, but it will prove probably more satisfactory from the military standpoint.

### RATIONS IN INSTITUTIONS.

Poor-Law institutions are to be rationed. A circular has been sent by the Local Government Board to the Boards of Guardians stating that the new rationing scale issued by the Ministry of Food must be substituted for the previous scale of the Food Controller so far as the dietaries of certain inmates are concerned. Commenting upon this, the Birmingham Guardians say that the new scale deals with bread, cereals, meat, butter and margarine and other fats, and sugar. As regards tea, butter, or margarine, the rationing scale drawn up by the Local Food Committee provides for the issue of one ounce of tea and four ounces of butter or margarine per head per week for the month of January. The issues of butter or margarine can be supplemented under the Ministry of Food scale by the issue of other fats so long as the total does not exceed ten ounces per head per week. It is interesting to note that authority is now given for a smaller allowance than the prescribed ration of potatoes and other vegetables to be issued to the inmates in the first instance. The inmates, however, will receive the full allowance if they are able to consume it. This is similar to the arrangements with regard to bread which have been in operation and found to be very economical for some years. The head officials of the Guardians' institutions have been requested to effect the necessary alterations in the dietaries and to forward a full report at an early date as to the changes made and any difficulties which have arisen in putting into operation the new scale. Experiments have been made with the making of bread with a percentage of potatoes added to the flour, and very satisfactory results have been obtained.

### THE LATEST REPORT ON THE POOR LAW.

The thorny question known as Poor-Law reform has produced probably more reports than any other. The latest is that presented by the sub-committee which has been considering, under the Reconstruction Committee, what changes in local government are desirable after the war. Under the chairman, Sir Donald Maclean, Lord George Hamilton and Mrs. Sidney Webb, the respective authors of the majority and minority reports of the Poor-Law Commission, are among the members. This being so, it is noteworthy that Mrs. Webb's views seem to have prevailed, for this report, like that of the minority, advocates the abolition of the Guardians, and the transference of their duties. It is alleged, for we cannot admit that the result is satisfactory, that the Insurance Act has secured medical relief, maternity benefit, and treatment for tuberculous cases, that medical inspection is now carried out by the officers of the education committees, and that municipalities are now charged with dealing with unemployment, and also have their own infectious diseases hospitals and asylums. The multiplication of authorities has certainly been one of the chief causes of overlapping, and there is something to be said for entrusting with relief in its various forms bodies which are free from the shackles of a Poor Law which has sacrificed the self-respect of most of those assisted by it.

### "THE SEARCHLIGHT."

The current number of the *Gazette* of the 2nd Western General Hospital, Manchester, is mainly remarkable for a contribution by an American officer, who gives his impressions. The weather, the food, the slang, the preoccupation with bridge, all come in for comment, and Chatham's hospital is mentioned by the writer as one of the old buildings which Americans wish to see. There is a welcome collection of notes from former patients and members of the staff who are now widely scattered, besides a plentiful supply of light verse. We wish that more American impressions might find their way into these *Gazettes*, for Englishmen know better their fellow-countrymen's impressions of America than their cousins' sensations upon arriving in England. No doubt these will come in time, but meanwhile the *Searchlight* has set a commendable example.

### THIS WEEK'S DRUG MARKET.

Business is quiet and price changes are few. The main item of importance is potassium bromide, which is extremely scarce and likely to become dearer; this applies to all the bromides. Aspirin is again rather lower. A fair business has been done in quinine, and the price tendency is upwards. Antifebrin and hexamine are dearer.

